# A Novel Intelligent Indicator Film: Preparation, Characterization, and Application

**DOI:** 10.3390/molecules28083384

**Published:** 2023-04-11

**Authors:** Bing Han, Peifeng Chen, Jiaxuan Guo, Hongliang Yu, Shaojing Zhong, Dongmei Li, Chunhong Liu, Zhibiao Feng, Bin Jiang

**Affiliations:** Department of Chemistry, College of Arts and Sciences, Northeast Agricultural University, Harbin 150030, China

**Keywords:** whey protein isolate nanofibers, anthocyanin, edible film, food packaging, food spoilage

## Abstract

The development of intelligent indicator film that can detect changes in food quality is a new trend in the food packaging field. The WPNFs-PU-ACN/Gly film was prepared based on whey protein isolate nanofibers (WPNFs). Anthocyanin (ACN) and glycerol (Gly) were used as the color indicator and the plasticizer, respectively, while pullulan (PU) was added to enhance mechanical properties of WPNFs-PU-ACN/Gly edible film. In the study, the addition of ACN improved the hydrophobicity and oxidation resistance of the indicator film; with an increase in pH, the color of the indicator film shifted from dark pink to grey, and its surface was uniform and smooth. Therefore, the WPNFs-PU-ACN/Gly edible film would be suitable for sensing the pH of salmon, which changes with deterioration, as the color change of ACN was completely consistent with fish pH. Furthermore, the color change after being exposed to grey was evaluated in conjunction with hardness, chewiness, and resilience of salmon as an indication. This shows that intelligent indicator film made of WPNFs, PU, ACN, and Gly could contribute to the development of safe food.

## 1. Introduction

Edible films as innovative packaging materials have become a hot topic in recent years. The edible films can be used to increase food stability by limiting moisture, lipid, volatiles, and gas exchange between the meal and the surrounding environment [1]. It has been suggested that edible films prepared by natural macromolecules such as proteins, polysaccharides, and lipids can be used as potential substitutes for packaging materials [2,3,4]. Natural proteins such as soy protein, zein, casein, and whey protein [5], as the main components of edible film, need to be denatured to form a more functional edible film [6]. However, most of the protein edible film materials are made of a single component, and there is a lack of innovation. Whey protein isolate nanofibers (WPNF) have the potential to be an edible film due to their ability of adsorption and unfolding on the interface [7]. Due to the edible film of a single substance having certain functional defects, composite films are suggested to avoid the disadvantages of single-component films. Mixing with different substances will complement the functions of the films and make the composite films more ideal in terms of functional properties. Protein–polysaccharide complexes can form networks and composite edible films has better mechanical properties [8]. Protein–lipid complexes can produce composite edible films with good water-resistance properties [9].

In recent years, intelligent food packaging has attracted significant attention [10]. Intelligent packaging monitors food quality and gives status information which could contribute to food safety. Therefore, it must be nontoxic and sensitive to deterioration factors [11]. Anthocyanin (ACN), a natural pigment, has the potential to be used as an indicator in intelligent packaging. In fact, many functional materials used for food packaging (e.g., antioxidative, bacteriostatic, pH-sensitive, and time-temperature indicators) are achieved by the addition of ACN [12].

Mulberry is a mostly red-purple or black oval-shaped polygamous fruit, which is widely grown in Central Asia and Europe [13]. Mulberry, which is enriched with resveratrol, flavonoids, anthocyanins, and polysaccharides, can be used as both medicine and food. Mulberry possesses many functional properties including immunological regulation, antioxidant, and anti-tumor effects [14]. It was proved that mulberry extract has significant protective effect on hyperlipemia and diabetes. Mulberry extract (ME) can inhibit the expression of inflammation-related factors, thus weakening the pro-inflammatory response and playing an anti-inflammatory role [15]. Anthocyanin (ACN) in mulberry (mainly cornetin) is a water-soluble natural pigment that appears red, purple, and blue in plants. ACN is harmless to the human body and has good antioxidant, anti-inflammatory, and anticancer effects [16]. Changes in pH value will change the structure of ACN, causing ACN to exhibit different colors [17]. In the preparation of protein–lipid composite films, the dissolution needs to be facilitated with the assistance of emulsifiers because ACN and WPNFs are immiscible. WPNFs have strong emulsifying properties, and can make the solution stable without adding emulsifiers, so they exhibit a good application prospect in the field of food packaging [18]. Furthermore, WPNFs also have antioxidant properties, which can make the color of ACN more stable.

In this study, an intelligent indicator edible film which could be used for food packaging and food spoilage was prepared. Several key characterization methods were used to determine the microstructure and physicochemical properties of the edible film. The antioxidant activity of edible film-forming solutions and color protection of WPNFs on ACN were also evaluated. Finally, salmon was chosen as the indicator, and the pH and texture of salmon at different storage times and corresponding colors of edible film were analyzed.

## 2. Results and Discussion

### 2.1. Identification of ACN

The chromatographic and spectral data of ACN are exhibited in Table S1, and Figure S1 shows the mass spectrum and structure of each ACN from mulberry. Peak 1 (*t_R_* = 2.684 min) had a molecular ion peak at *m*/*z* 611 and a fragment ion peak at *m*/*z* 287. Peak 1 was identified as Cyanidin-3-sophoroside. For peak 2 (*t_R_* = 3.046), there was a molecular ion peak at *m*/*z* 449 and a fragment ion peak at *m*/*z* 287, indicating that the structure of peak 2 was composed of a centaurin parent nucleus and a molecule of glucose [19,20]. Peak 2 was identified as cyanidin-3-glucoside [21,22,23]. Since the molecular ion peak of peak 3 (*t_R_* = 3.231 min) was *m*/*z* 595 and the fragment ion peak was *m*/*z* 287, it could be inferred that the structure of peak 3 was composed of a centaurin parent nucleus, one molecule of glucose, and one molecule of rhamnose. Peak 3 was identified as cyanidin-3-rutinoside [24,25]. Peak 4 (*t_R_* = 3.727 min) had a molecular ion peak at *m*/*z* 463 and a fragment ion peak at *m/z* 301, indicating that the structure of peak 4 contained paeonidin parent nucleus and one molecule of glucose; peak 4 was identified as peonidin-3-glucoside [21,22,23]. In summary, there were four different structures of ACN from mulberry.

### 2.2. PH Sensitivity of ACN

In nature, ACN is widely distributed in fruits and vegetables. The same ACN may appear in different colors due to the differences in pH of the cellular environment. Figure S2 shows that the color of ACN changed significantly in different pH buffers. The color variation of ACN in the buffer was red/pink at pH 2.0–6.0; light purple at pH 7.0; dark purple at pH 8.0; purple/blue-purple at pH 9.0–11.0; and gray-purple at pH 12.0. Under strong acidic condition, ACN was presented in the form of a pyranoid cation and its solution was red. Under slightly acidic condition, ACN was mainly presented in the form of colorless chalcone. With the increase of chalcone content, the color of solution gradually became lighter [26]. However, when the pH was 6.0, the solution was dark pink. This might be due to an increase in pH of the buffer, and the content of quinonyl also increased, resulting in a superposition of colors. Under weakly alkaline condition, ACN was mainly presented in the form of blue quinonyl, and with the increase of quinonyl, the solution color turned blue [25,27]. Figure S3 shows the UV-vis spectra (wavelength range 400–800 nm) of ACN in different buffer solutions (pH 2.0–12.0). The maximum absorption peak of the ACN solution was redshifted from 520 nm to 580 nm. The color change of the ACN solutions and the corresponding redshift of the maximum absorption peaks were mainly caused by the structural transformation of ACN in different pH buffer solutions [28,29]. Therefore, different pH values could cause changes in the structure of ACN, which could result in different colors of ACN.

### 2.3. Antioxidant Activity of the Film-Forming Solutions

The antioxidant activity of the film-forming solutions and ACN was evaluated by ABTS free radical scavenging test. The phenolic hydroxyl groups on ACN were easily oxidized and bound to reactive oxygen species or other free radicals [30,31]. Figure S4 indicated that ACN had antioxidant activity (60.75 ± 1.61%). Compared with WPI-PU/Gly film-forming solution, WPNFs-PU/Gly film-forming solution exhibited higher ABTS^+^ free radical scavenging activity, which might be due to the antioxidant activity of WPNF itself [4,32]. After the addition of ACN, the ABTS^+^ free radical scavenging activity of all film-forming solutions were increased significantly. Compared to the film-forming solutions without adding ACN, the antioxidant activity of WPI-PU-ACN/Gly and WPNFs-PU-ACN/Gly film-forming solutions had increased by 23.66% and 36.81%, respectively. WPNFs-PU-ACN/Gly film-forming solution showed higher antioxidant activity, which might be due to the combined action of the antioxidant activity of ACN and WPNFs [33,34]. At the same time, the higher antioxidant activity of WPNFs-PU-ACN/Gly film-forming solution could also protect ACN from fading due to being oxidized.

### 2.4. Color Protection of ACN by WPNFs

**Figure 1:**
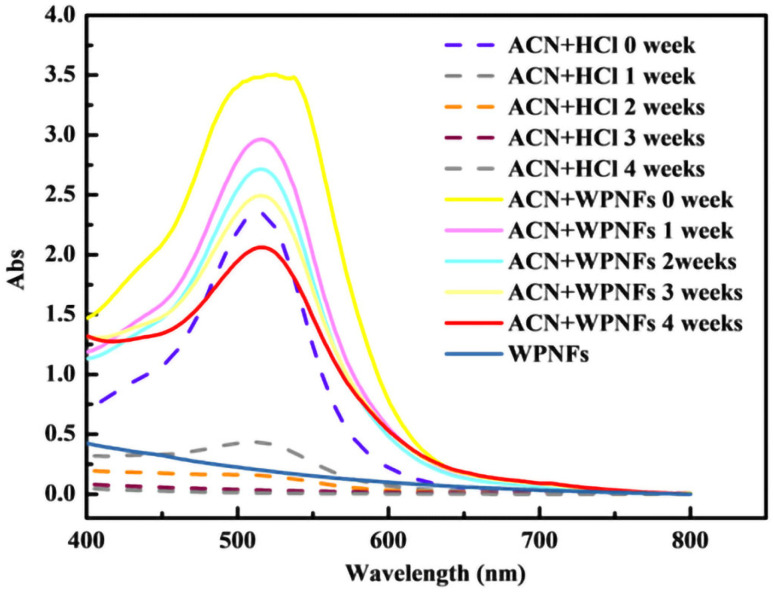
UV-vis spectra of ACN in different solutions (pH = 2); the wavelength ranges: 400–800 nm.

ACN has poor stability and is prone to fade due to oxidation. The stability of ACN was affected by many different factors, such as content, structure, temperature, pH, and light intensity. Therefore, the stability of ACN could be improved by preserving it at low temperature or away from light or changing its structure. Figure 1 shows the UV-vis spectra of ACN in hydrochloric acid solution with pH of 2.0 (dashed line) and WPNFs solution with pH of 2.0 (solid line). No significant peak was observed on the curves of WPNFs solutions near 520 nm. With the increase of time, the maximum absorbance of ACN in WPNFs solution decreased more slowly compared with the control group. This phenomenon might be due to the cross-linking of WPNF and ACN or the change in turbidity of solution. The hydroxyl on the B ring of ACN structure was bound to WPNFs by hydrophobic forces and hydrogen bonds, which protected the color of ACN. In addition, good antioxidant activity of WPNFs could also protect ACN from fading by oxidation.

### 2.5. Water Contact Angle Analysis of the Edible Films

**Figure 2:**
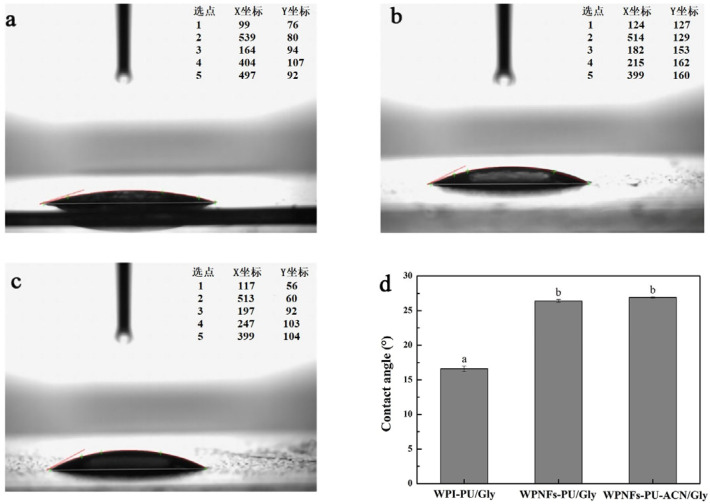
The drop shape and water contact angle of edible films; lateral photograph of water drops on the edible films: (**a**) WPI-PU/Gly edible film, (**b**) WPNFs-PU/Gly edible film, (**c**) WPNFs-PU-ACN/Gly edible film, and (**d**) comparison of water contact angle of the surface of the edible films. Different letters (**a**,**b**) represent significant differences (*p* < 0.05).

The most visual indicator of hydrophilicity or hydrophobicity of films was the water contact angle determination [35]. It was reported that the film was hydrophobic when the water contact angle θ > 65° and hydrophilic when θ < 65° [36]. When the film has a smaller water contact angle, the film is more hydrophilic. Conversely, the film is more hydrophobic. Figure 2 shows that all the water contact angles of edible films were less than 65°, which indicated that all the edible films were hydrophilic. Compared with the water contact angle of WPI-PU/Gly edible film (16.6° ± 0.4°), the water contact angle of WPNFs-PU/Gly edible film was slightly larger (26.4° ± 0.2°). According to a similar study, the well-organized β-sheet structures created during the production of WPNFs could result in an increased hydrophobicity [37]. The addition of ACN slightly increased the water contact angle of the edible film (26.9° ± 0.1°), which might be attributed to the interaction between ACN and WPNFs. Although the WPNFs-PU-ACN/Gly edible film was more hydrophobic than other edible films, all the edible films were still hydrophilic. When the aqueous solution encountered the surface of WPNFs-PU-ACN/Gly edible film, the liquid could diffuse well on the surface of WPNFs-PU-ACN/Gly edible film, which enabled the WPNFs-PU-ACN/Gly edible film to fit better on the food surface. In fact, edible film with excessive hydrophilic will dissolve the ACN, which could cause ACN to leak, affecting the appearance of the food, while edible film that is too hydrophobic would affect the determination of color. Therefore, it is very necessary to choose a suitable hydrophilicity and hydrophobicity for the color indicator edible film.

### 2.6. Microstructure of the Edible Films

**Figure 3:**
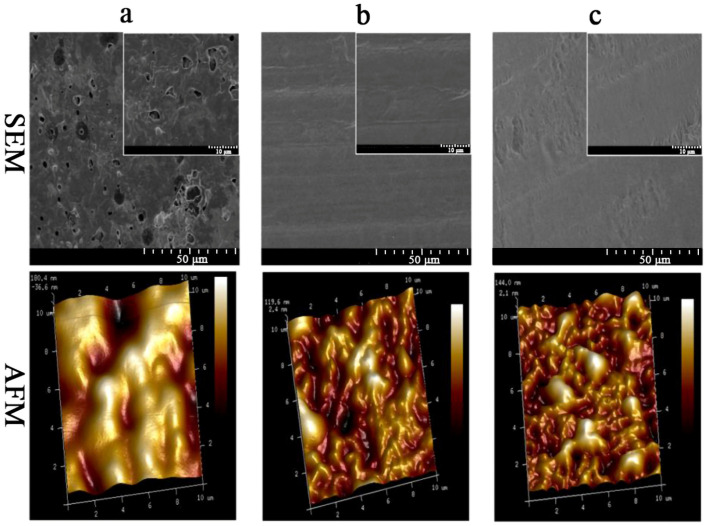
SEM and AFM micrographs images of edible films: (**a**) WPI-PU/Gly edible film, (**b**) WPNFs-PU/Gly edible film, and (**c**)WPNFs-PU-ACN/Gly edible film.

SEM was used to evaluate the microstructure, uniformity, and surface smoothness of the edible films. The uniformity and surface smoothness of the edible films reflect their resistance to oxygen and carbon dioxide [38]. The surface microstructure of the edible films is shown in Figure 3. The surface of WPI-PU/Gly edible film was rough (Figure 3a), on which many protein particles and pores could be observed. In contrast, the surface of the WPNFs-PU/Gly edible film was uniform, smooth, and continuous without pores (Figure 3b). The surface of WPNFs-PU-ACN/Gly edible film was relatively smooth (Figure 3c). Compared with WPNFs-PU/Gly edible film, the surface of WPNFs-PU-ACN/Gly edible film was slightly uneven, which might be related to the addition of ACN.

The surface morphology and roughness of edible films were evaluated by AFM. The surface of the WPI-PU/Gly edible film was rough and contained some pores (dark areas) with a large undulating range (Figure 3a). The average roughness of WPI-PU/Gly edible film surface was 64.3 nm. Compared with the WPI-PU/Gly edible film, the surface of the WPNFs-PU/Gly edible film was relatively flat with an average surface roughness of 32.9 nm (Figure 3b), and the roughness of the WPNFs-PU-ACN/Gly edible film was slightly raised with an average surface roughness of 39.7 nm (Figure 3c). The AFM results of the edible films were consistent with the results of SEM.

### 2.7. PH Sensitivity Analysis of Edible Films

**Figure 4:**
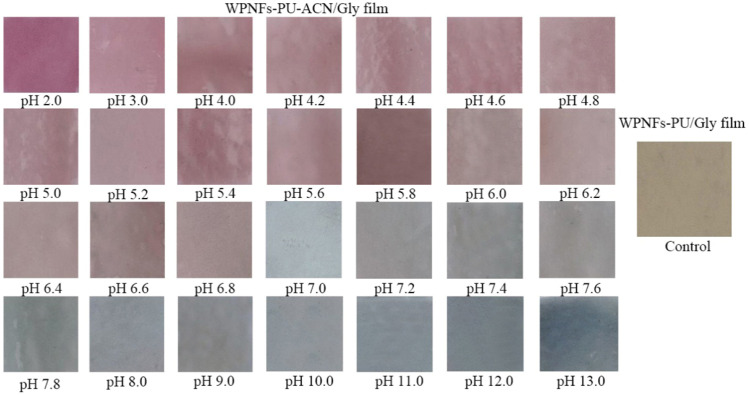
Color of WPNFs-PU-can/Gly edible film after being immersed in different buffer solutions (pH 2.0 to 13.0) for 15 min.

The pH sensitivity of the edible films was measured by the color change of the edible films in different pH buffer solutions (Figure 4). The color of WPNFs-PU/Gly edible film did not change with pH values. However, WPNFs-PU-ACN/Gly edible film was sensitive to pH. The color of WPNFs-PU-ACN/Gly edible film changed significantly in different pH buffer solutions from dark pink to gray as the pH increased. This was similar to the color change of ACN in different pH buffer solutions. The edible films prepared by Kurek et al. [39] with agar/potato starch as substrate and purple potato extracts as color indicator were sensitive to pH. In addition, it has been reported that the films were prepared by adding cabbage extract [40] and blackberry extract [41] to the substrate, and the ACN contained in the two could be used as a color indicator. WPNFs-PU-ACN/Gly edible film had good pH sensitivity, and the color of the edible film was more stable due to the color protection of WPNFs, which did not fade easily. Therefore, it could be used as a pH indicator film to roughly indicate the quality of food in the future.

### 2.8. Quality Assessment of Salmon

#### 2.8.1. PH and TPA Changes of Salmon during Storage

**Figure 5:**
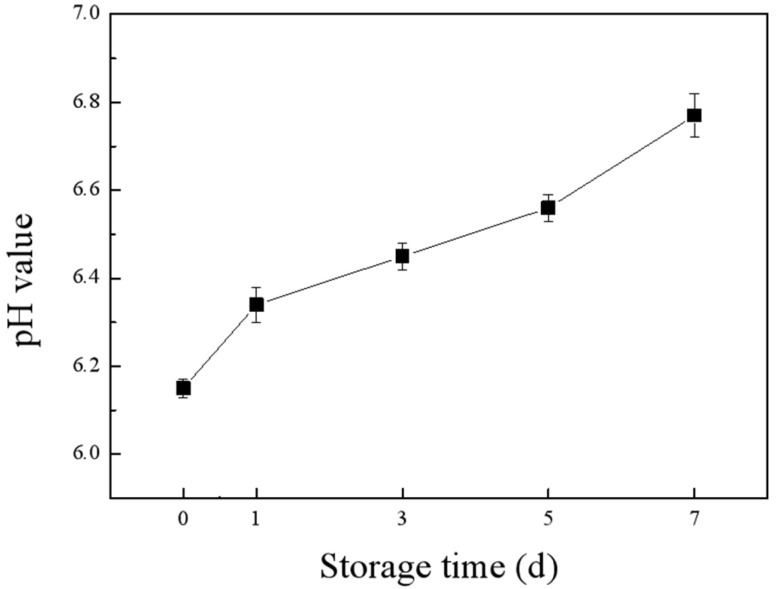
The pH changes of salmon during storage at room temperature.

Fish contains a variety of unsaturated fatty acids, proteins, free amino acids, and endogenous enzymes, and it is susceptible to being oxidized and degraded by microorganisms. We used salmon as an indicator and measured its pH changes while stored at 25 °C for 7 days. As shown in Figure 5, the pH of the salmon increased gradually with the increase of storage time. The protein in the salmon was broken down and produced ammonia compounds and other alkaline substances, which increased the pH. This was consistent with the results of Huang et al. [42], who found that the pH of salmon increased with the prolongation of its storage period at 25 °C. Texture is a valuable quality indicator that influences the sensory and functional characteristics of fish. In our study, hardness, chewiness, and resilience were used as indexes to evaluate the texture characteristics. The TPA of salmon from Table S2 showed that the hardness, resilience, and chewiness of salmon all decreased with the increase of storage time. During the storage process, salmon gave off a slight spoilage odor and produced a small amount of juice from the third day onwards. As the storage period extended, the odor of spoilage became intense, and the surface color of salmon became significantly lighter.

#### 2.8.2. Color Response of WPNFs-PU-ACN/Gly Edible Film during Salmon Storage

**Figure 6:**
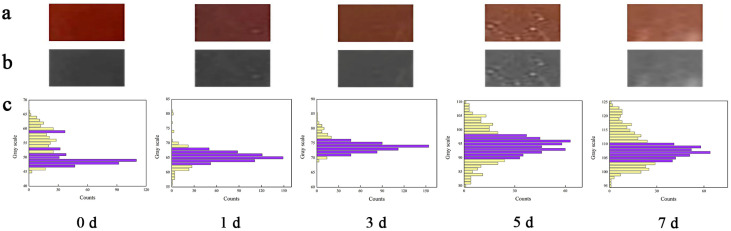
The color change of WPNFs-PU-ACN/Gly edible film and the corresponding grayscale range at 25 °C for different storage times (0, 1, 3, 5, and 7 days): (**a**) actual color change of WPNFs-PU-ACN/Gly edible film, (**b**) color change of WPNFs-PU-ACN/Gly edible film after grayscale, (**c**) grayscale range corresponding to the color change of WPNFs-PU-ACN/Gly edible film.

When the spoilage degree of salmon is relatively slight, it is difficult to determine whether it is spoiled (or the degree of spoilage) based on its color and smell. Therefore, we could predict the spoilage degree of salmon by the color change of WPNFs-PU-ACN/Gly edible film. The photographs of color change of WPNFs-ACN/PU/Gly edible film during storage (Figure 6a) were grayed using Photoshop software, and grayscale was used instead of RGB to describe the color information of the photographs. Color image grayscaling is essentially a dimensionality reduction operation; that is, the use of a one-dimensional grey space transformed by some transformation relation to represent the data information in the original three-dimensional color vector space [43]. In a greyscale image, because there is no more color information, the contrast between pixel dots directly reflects the contrast information of the original color image [44]. The color change of WPNFs-ACN/PU/Gly edible film during storage after grayscale processing was shown in Figure 6b. The color of WPNFs-ACN/PU/Gly edible film gradually became lighter, and the gray value gradually increased with the salmon storage time increasing. To further obtain the grayscale range accurately, Matlab software was used to analyze the number of pixels corresponding to all grayscales, and the corresponding grayscale range of WPNFs-ACN/PU/Gly edible film in salmon storage time was obtained. We assumed that if the number of pixels in the corresponding time period was less than 30, it did not match the characteristics of the grayscale in that time period. As shown in Figure 6c, the grayscale ranges of WPNFs-ACN/PU/Gly edible film corresponding to salmon stored for 0, 1, 3, 5, and 7 days were 47–54, 63–68, 71–76, 90–98, and 104–110, respectively. Therefore, the grayscale value of WPNFs-ACN/PU/Gly edible film could be used to evaluate the spoilage degree of salmon, which is intuitive, effective, and simple. Meanwhile, WPNFS-ACN/PU/Gly edible film is biocompatible, which will not affect the safety of the food.

## 3. Materials and Methods

### 3.1. Materials

Whey protein isolate (WPI, protein content > 91.5%) was acquired from Baiwei Biotechnology Co., Ltd. (Hebei, China). Glycerol (Gly) and pullulan (PU) were obtained from Aladdin Reagent Co. (Shanghai, China). Additionally, 2,2′-azinobis (3-ethylbenzothiazoline-6-sulfonic acid) diammonium salt (ABTS) and other reagents were purchased from Solarbio (Beijing, China). Salmon and mulberry were purchased from the local market (Harbin, China).

### 3.2. Extraction and Characterization of ACN from Mulberry

#### 3.2.1. Extraction of ACN

The mulberry was broken and immersed in ethanol-water-hydrochloric acid solution (50:50:0.5, *v*/*v*/*v*) at a ratio of 1:10 (*w*/*v*). The mixture was shaken by ultrasonic shaking (JY92-2D, SCIENTZ, Ningbo, China) at 30 °C for 2 h and then filtered. The filtrate was placed on a rotary evaporator (N-110, EYELA, Tokyo, Japan) at 35 °C to concentrate the sample to remove some of the ethanol. Finally, the anthocyanin concentration solution was freeze-dried by a vacuum freeze dryer (Vaco 5-80, ZIRBUS, Berlin, Germany) for 2 d [45]. The ACN extracts from mulberry were obtained.

#### 3.2.2. Purification of ACN

The extracts of ACN from mulberry were loaded on an AB-8 microporous resin column. The column was washed with 1% (*v*/*v*) HCl after ACN was completely adsorbed. Then 70% (*v*/*v*) ethanol containing 1% (*v*/*v*) HCl was used to recover the ACN. Residual solvent was removed by rotary evaporator at 40 °C. The reddish black ACN powders were obtained by freeze-drying.

#### 3.2.3. Identification of ACN

ACN from mulberry was identified by UHPLC-MS/MS, which was performed by a mass spectrometer (5600+ Q-TOF, AB SCIEX, Boston, MA, USA) coupled to an UHPLC (ExionLC AD, AB SCIEX, Boston, MA, USA) system equipped with a C18 column (2.1 × 100 mm, 1.7 µm, ACQUITY UPLC BEH, Waters, Boston, MA, USA). Elution was carried out using a gradient system with 0.1% (*v*/*v*) formic acid (mobile phase A) and acetonitrile (mobile phase B) as follows: the linear gradient began with 5% B and ended at 15% B after 10 min, and the linear gradient increased to 90% B in the following 5 min, then held at 95% B for 5 min. Finally, the mobile phase composition was immediately changed back to 5% B and held at 5% B for 5 min. The flow rate was 0.4 mL/min. The column temperature was 30 °C and the injection volume was 5 µL. The detection wavelength of UHPLC was 512 nm. Spectra of MS were recorded in positive ion mode between *m*/*z* 100 and 1000. The main parameters of MS were as follows: ion spray voltage floating, 5500 V; aerosol gas, 50 psi; auxiliary heating gas, 50 psi; curtain gas, 35 psi; temperature, 550 °C; declustering potential, 90 V. The collision energies of MS and MS^2^ were set to 10 V and 35 V [46].

#### 3.2.4. PH Sensitivity of ACN

ACN 2 mg was dissolved in 20 mL different buffer solutions (pH 2–13). UV/vis spectra of the solutions were determined by an ultraviolet spectrophotometer (UV-2550, Shimadzu, Kyoto, Japan) in the wavelength range of 400–800 nm [47]. Ultrapure water acted as a blank calibration.

### 3.3. Preparation of the Film-Forming Solutions

The preparation method of WPI and WPNFs was reported in our previous study [48]. Briefly, WPI solution (5%, *w*/*v*) was prepared by dissolving WPI in ultrapure water. Then, HCl (3 mol/L) was added dropwise to the solution to adjust pH to 2.0 and stirred for 30 min at room temperature. The solution was centrifuged at 9000 rpm for 15 min at 4 °C (Z236HK Hermle, Wehingen, Germany). The above supernatant was vacuum filtered through a fiber membrane (0.45 µm, Aladdin, Shanghai, China) to remove undissolved proteins. The filtered solution was incubated at 80 ℃ for 10 h with 220 rpm constant magnetic stirring to obtain WPNFs solution. Gly (5% *w*/*v*) was mixed with the WPNFs solution and the solution was heated in a water bath at 80 °C for 30 min. After the solution was cooled, the WPNFs-PU-ACN/Gly edible film-forming solution was prepared by adding PU (0.5% *w*/*v*) and ACN (0.25%, *w*/*v*) to the above solution. In addition, WPI-PU-ACN/Gly edible film-forming solution was prepared by similar procedure in which WPNFs solution was replaced by WPI solution. WPI-PU/Gly and WPNFs-PU/Gly edible film-forming solutions were prepared without adding ACN.

### 3.4. Characterization of Edible Film-Forming Solutions

#### 3.4.1. Antioxidant Activity of the Film-Forming Solutions

First, 2 mL of ABTS solution (7.4 mmol/L) was mixed with 35 µL of potassium persulfate (140 mmol/L), and the mixed solution was stored in the dark for 14 h. Phosphate buffer solution (pH = 7.4) was used to dilute the above solution before use, and the absorbance at 734 nm was adjusted within 0.7 ± 0.02. 50 µL of the film-forming solutions and 3 mL diluted ABTS solution was mixed and stored in the dark for 0.5, 1, 1.5, 2, 2.5, and 3 min, respectively. The absorbance of the above solutions was measured at 734 nm [49,50].

#### 3.4.2. Color Protection of WPNFs on ACN

First, 4.0 mg of ACN was added to 20 mL of WPNFs solution, and the sample solution was stored at room temperature (20–25 °C) for 4 weeks. The UV/vis spectrum of the sample was measured in a wavelength range of 400–800 nm each week. The control was HCl solution (pH 2) with 4 mg ACN. The WPNFs solution and ultrapure water were used as a blank calibration for sample and control, respectively.

### 3.5. Preparation of Edible Films

First, 20 mL of film-forming solution was evenly coated on the polypropylene sheet and stood for 3 h to remove air bubbles. The edible films were obtained by drying in a constant temperature drying oven at 40 °C for 16 h [51,52].

### 3.6. Functional Properties and Evaluation of Edible Films

#### 3.6.1. Water Contact Angle of Edible Films

Water contact angle of edible films was measured by a water contact angle meter (XG-CAM, Sunzern Instrument, Shanghai, China). The analysis was conducted using the ADSA-Real Drop method and the contact angle analysis software CAST 3.0. The edible films were cut into 10 × 10 cm and fixed to glass slides as flat as possible. A droplet of water was added to the surface of the edible film. The water contact angles were measured from 0 to 180° with accuracy of ±0.1°.

#### 3.6.2. Scanning Electron Microscope (SEM) Analysis of Edible Films

The edible films were cut out and coated with a fine gold layer. The micromorphology of the edible films was captured by SEM (S-3400N, Hitachi, Tokyo, Japan). All the edible films were examined using an accelerating beam at a voltage of 5 kV.

#### 3.6.3. Atomic Force Microscope (AFM) Analysis of Edible Films

The microstructure and surface roughness of the edible films were determined by AFM (Dimension Fastscan, Bruker, Boston, MA, USA) with tap mode. Then, 20 µL of film-forming solution was applied uniformly on a 5 µm × 5 µm silicon wafer by using a rotary coater with 3000 r/min. The images were processed and analyzed using NanoScope Analysis software (v1.50, Bruker, Boston, MA, USA).

#### 3.6.4. PH Sensitivity of Edible Films

The edible films were immersed in different buffer solutions (pH 2–13) for 15 min and then photographed [53]. The buffer solutions here were 1 mol/L aqueous lactic acid (pH 2.0, 3.0, 4.0, 5.0, and 6.0) and 1 mol/L aqueous sodium phosphate monobasic buffer (pH 7.0, 8.0, 9.0, 10.0, 11.0, and 12.0) [52]. Twenty different points were randomly collected in each image, and R, G, and B values for each point were obtained by using Adobe Photoshop software (2019cc, Adobe Systems, Los Angeles, CA, USA).

### 3.7. Quality Assessment of Salmon

#### 3.7.1. Detection of Salmon Freshness

Freshly peeled salmon was cut into small cubes of 1.5 × 1.5 × 1.0 cm^3^, and each piece weighed approximately 15 g. The WPNFs-ACN/PU/Gly film was cut into small pieces, stuck to the surface of the salmon, and sealed with plastic wrap in a petri dish. The salmon was observed at room temperature for 7 days [41,52]. The color change of WPNFs-ACN/PU/Gly edible film was photographed and recorded. Photographs were taken at the same time point, location, and light source.

#### 3.7.2. PH of Salmon

The salmon was chopped and homogenized at 20,000 rpm for 5 min. Then, 10 times the mass of potassium chloride solution (0.1 mol/L) was added and homogenized again at 20,000 rpm for 5 min. A pH-meter (FE-20, Mettler Toledo, Zurich, Switzerland) was employed to determine the pH of the homogenate.

#### 3.7.3. Determination of Texture Profile Analysis (TPA)

The method is mainly based on Yu [54] and Aguilera Barraza [55] with modifications. The TPA of salmon was measured by a texture analyzer (TA.XT PlusC, Stable Micro Systems, London, UK). A P5 probe was used to press the sample blocks to achieve 50% compression relative to the block height in a two-cycle compression test. The samples were measured with a 5 g trigger force at a pretest speed of 3 mm/s, test speed of 1 mm/s, and a post-test speed of 1 mm/s.

### 3.8. Statistical Analysis

All the experiments were repeated three times, and the results were expressed as mean and standard deviation. The significant analysis of variance was carried out by SPSS 20.0 (Chicago, IL, USA), and the significance of difference (*p* < 0.05) was evaluated by Duncan’s multiple range test. The spatial analysis was performed using Matlab (2016a, MathWorks, Natick, MA, USA).

## 4. Conclusions

In this study, an intelligent indicator edible film was successfully prepared. Compared with WPI-PU-ACN/Gly film-forming solution, WPNFs-PU-ACN/Gly film-forming solution has higher antioxidant activity. The antioxidant activity could protect ACN from being destroyed. The WPNFs-PU-ACN/Gly edible film showed different colors in different pH buffer solutions. With perishable salmon as an indicator, the spoilage degree of salmon could be easily and conveniently evaluated by the color change of WPNFS-PU-ACN/Gly edible film after grayscale. The research indicated that WPNFs-PU-ACN/Gly edible film has great potential application in the food industry.

## Data Availability

Data are available within the article.

## Supplementary Materials

The following supporting information can be downloaded at https://www.mdpi.com/article/10.3390/molecules28083384/s1, Figure S1: Mass spectrum and structure of each ACN from black rice; Figure S2: Color variations of ACN from mulberry in different buffer solutions (pH 2.0 to 12.0); Figure S3. UV-vis spectra of ACN in different buffer solutions (pH 2.0 to 12.0); Figure S4. ABTS radical-scavenging activity of different edible film-forming solutions. Different letters (a–d) represented significant differences (*p* < 0.05); Table S1: Chromatographic and spectral data of can from mulberry; Table S2: Differences in texture (hardness, chewiness, and resilience) of salmon cubes at different storage times (0, 1, 3, 5, 7 days).
