# Supplementary material for: A Novel Intelligent Indicator Film: Preparation, Characterization, and Application
Source: Molecules. 2023 Apr 11;28(8):3384. doi: 10.3390/molecules28083384 (PMC10143919; doi:10.3390/molecules28083384)
Supplement: Supplementary file 1 [file molecules-28-03384-s001.zip › molecules-2300084-supplementary.pdf]

A Novel Intelligent Indicator Film: Preparation, Characterization, and Application

Bing Han, Peifeng Chen, Jiaxuan Guo, Hongliang Yu, Shaojing Zhong, Dongmei Li, Chunhong Liu, Zhibiao Feng * and Bin Jiang *

Department of Chemistry, College of Arts and Sciences, Northeast Agricultural University, Harbin 150030, China; hanbingfor@163.com (B.H.); chenpeifeng20@163.com (P.C.); guojiaxuan77@163.com (J.G.); yuhongliang97@163.com (H.Y.); 17853510711@163.com (S.Z.); lidongmei@neau.edu.cn (D.L.); liuchunhong@neau.edu.cn (C.L.)

* Correspondence: fengzhibiao@neau.edu.cn (Z.F.); jiangbin@neau.edu.cn (B.J.); Tel.: +86-451-5519-02-22 (Z.F.); +86-451-5519-09-74 (B.J.)

Table S1. Chromatographic and spectral data of ACN from mulberry.

Peak NO.	Retention Time t_R (min)	[M]+ (*m/z*)	MS/MS (*m/z*)	Compound
1	2.684	611.1609	287.0550	Cyanidin-3-sophoroside
2	3.046	449.1083	287.0545	Cyanidin-3-glucoside
3	3.231	595.1662	287.0545	Cyanidin-3-rutinoside
4	3.727	463.1235	301.3706	Peonidin-3-glucoside

Table S2. Differences in texture (hardness, chewiness and resilience) of salmon cubes at different storage times (0, 1, 3, 5, 7 days).

Storage time (d)	Hardness	Chewiness	Resilience
0	447.424 ± 6.821 ^{e}	46.186 ± 2.482 ^{e}	0.082 ± 0.007 ^{d}
1	393.687 ± 7.193 ^{d}	36.221 ± 1.926 ^{d}	0.067 ± 0.006 ^{cd}
3	275.163 ± 7.838 ^{c}	20.353 ± 2.229 ^{c}	0.057 ± 0.004 ^{bc}
5	214.192 ± 6.203 ^{b}	14.437 ± 2.171 ^{b}	0.049 ± 0.005 ^{ab}
7	171.514 ± 7.384 ^{a}	10.657 ± 1.821 ^{a}	0.041 ± 0.008 ^{a}

Note: Different letters (a-e) represented significant differences ($p < 0.05$)

Additional figures

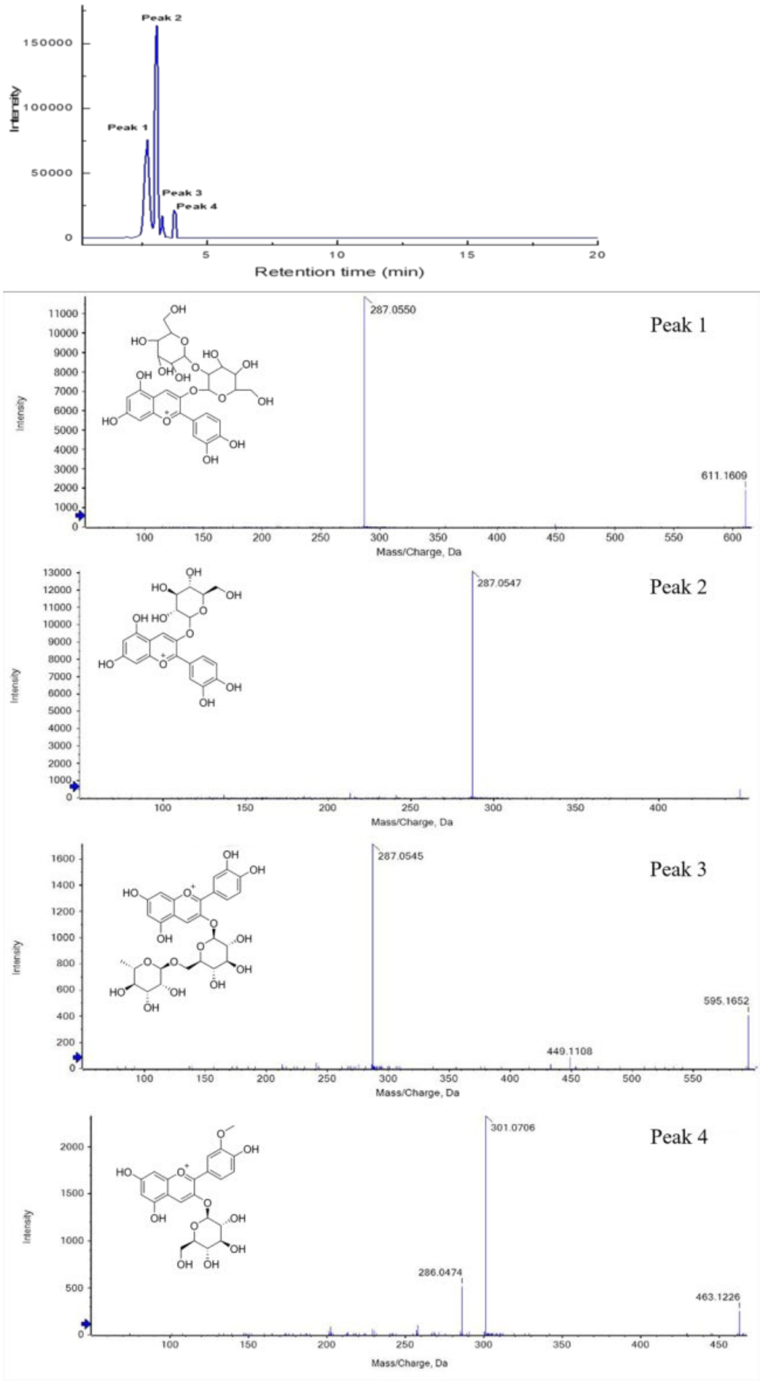

Figure S1. Mass spectrum and structure of each ACN from black rice.

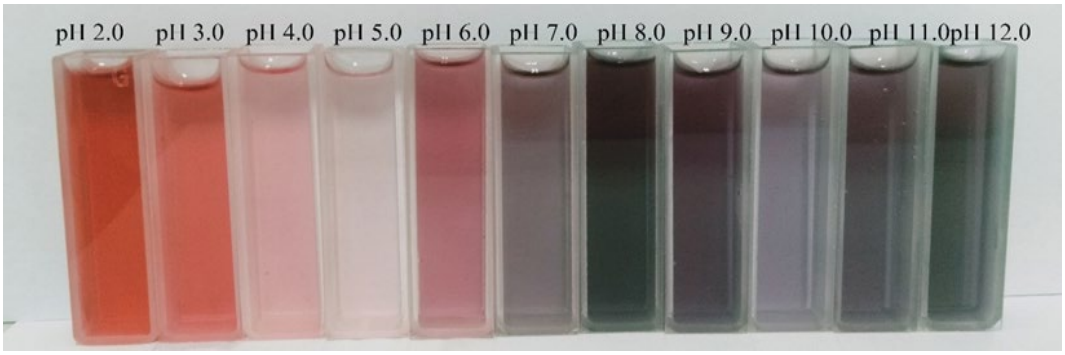

Figure S2. Color variations of ACN from mulberry in different buffer solutions (pH 2.0 to 12.0).

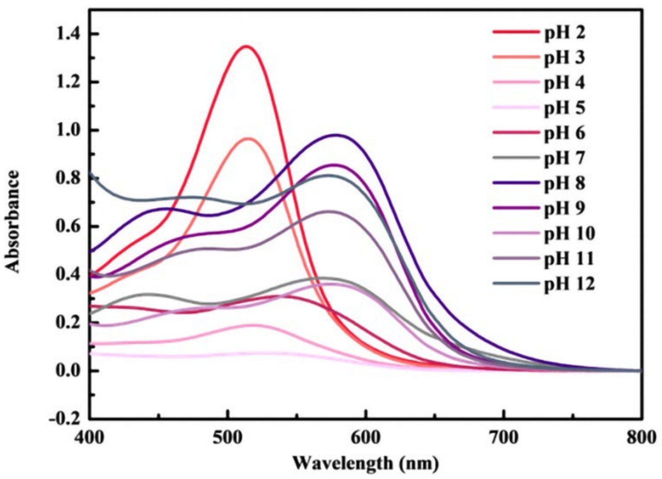

Figure S3. UV-vis spectra of ACN in different buffer solutions (pH 2.0 to 12.0).

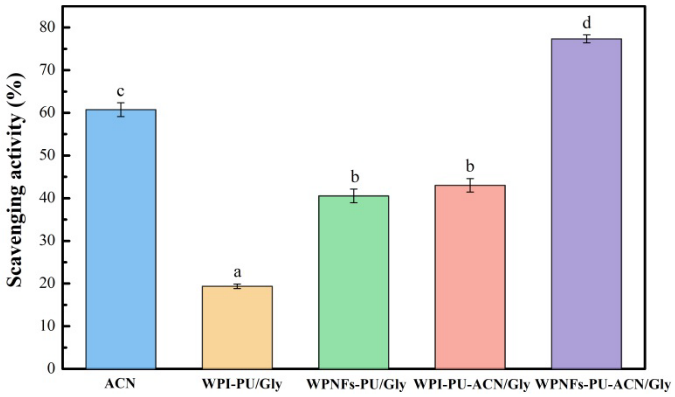

Figure S4. ABTS radical-scavenging activity of different edible film-forming solutions. Different letters (a-d) represented significant differences ($p < 0.05$).
